# An Integrated Transcriptomic and Proteomic Analysis Identifies Significant Novel Pathways for Henoch-Schönlein Purpura Nephritis Progression

**DOI:** 10.1155/2020/2489175

**Published:** 2020-06-19

**Authors:** Biao Xie, Wei Zhang, Qi Zhang, Qiuju Zhang, Yupeng Wang, Lin Sun, Meina Liu, Ping Zhou

**Affiliations:** ^1^Department of Biostatistics, Public Health College, Harbin Medical University, Harbin, Heilongjiang Province, China; ^2^Department of Pediatrics, The 2nd Affiliated Hospital of Harbin Medical University, Harbin, Heilongjiang, China

## Abstract

**Background:**

Although Henoch-Schönlein purpura nephritis (HSPN) is characterized by glomerular deposition of aberrantly glycosylated immunoglobulin A1 (IgA1), the underlying mechanism of HSPN progression has not yet been completely elucidated. In this study, we integrated transcriptomic and proteomic analyses to explore the underlying mechanism of HSPN progression.

**Methods:**

RNA sequencing and tandem mass tag- (TMT-) based quantitative proteomics were used to gain serum transcriptomic and proteomic profiles of patients with different types of HSPN (3 × type 1, 3 × type 2, and 3 × type 3). Student's *t*-tests were performed to obtain the significance of the differential gene expression. The clusterProfiler package was used to conduct the functional annotation of the DEGs for both Gene Ontology terms and Kyoto Encyclopedia of Genes and Genomes pathways.

**Results:**

A total of 2315 mRNAs and 30 proteins were differentially expressed between the different types of HSPN. 58 mRNAs and one protein changed continuously during HSPN development and are potential biomarkers for HSPN progression. The validation cohort (another 9 patients) confirmed the high-throughput results of the transcriptomic and proteomic analyses. A total of 385 significant pathways were related to HSPN progression, and four of them were closely related to clinical biochemical indicators and may play an important role in the progression of HSPN. Those pathways reveal that HSPN progression may be related to the inhibition of inflammation, promotion of apoptosis, and repair of renal injury.

**Conclusions:**

Four pathways were found to be closely related to HSPN progression, and it seems that HSPN progression is mainly due to the inhibition of inflammation, promotion of apoptosis, and repair of renal injury.

## 1. Introduction

Henoch-Schönlein purpura nephritis (HSPN) is the most serious complication of Henoch-Schönlein purpura (HSP) and occurs in approximately 30% of HSP pediatric patients within 4-6 weeks of the initial presentation [1–3]. According to the International Study of Kidney Disease in Children (ISKDC), HSPN can be divided into six subtypes (type 1 to type 6), with the first three being the most common in the clinic [4]. The prognosis of HSPN is closely related to its progression, with patients having possible decreased renal function, hypertension, hypoalbuminemia, and long-term renal sequelae in the later stages of HSPN [2]. In the clinic, there are targeted treatments for different HSPN types [4]. Exploring the mechanisms of HSPN progression may aid in finding effective diagnostic biomarkers and novel therapeutic targets [5]. Despite HSPN being mainly characterized by glomerular deposition of aberrantly glycosylated immunoglobulin A1 (IgA1), the underlying molecular mechanism of HSPN progression has not yet been completely elucidated [2, 6].

“Omics” have already been widely used in exploring complex diseases and have gathered further insight into the underlying mechanisms of disease development [7, 8]. Recently, there have been several HSP-related researches using genome-wide methods, including a thorough review published in *Autoimmunity Reviews* summarizing the genetic component associated with the pathogenesis of HSP [9]. Researchers in China have reported on the proteomic alterations between HSP and HSPN patients in the Chinese population using comparative proteomic analysis [10]. Additionally, we previously published a study that revealed three potential biomarkers associated with the progression of HSP to HSPN [11]. A single “omics” study however is only able to reveal the disease mechanism from one level. Disease progression is normally found to be closely related to differential gene expression, including mRNA and protein levels, and numerous studies have found significant correlations among different levels [12, 13]. Integration of different “omics” techniques facilitates the investigation of the possible mechanism from a systems biology point of view, providing a deeper understanding than any single “omics” study could do alone [14]. Previously, a study from China reported differential expression of long noncoding RNAs and mRNAs between children with HSPN and healthy children [15]; however, this study lacked proteomic data. Protein aggregation is known to be fundamental to HSPN pathogenesis, and the roles of abnormal proteins in HSPN are widely discussed [1–3]. To date, no study has explored HSPN with an integrated approach combining proteomic profiling and transcriptomics. Moreover, no previous studies have taken HSPN classification into consideration.

In this study, a comprehensive transcriptomic and proteomic analysis of HSPN patients' serums using RNA sequencing (RNA-seq) and tandem mass tag- (TMT-) based quantitative proteomics was performed. We aimed to identify mRNAs and proteins differentially expressed between different types of HSPN. Pathway enrichment analysis conducted on those differentially expressed mRNAs and proteins (DEGs) revealed pathways associated specifically with HSPN progression. These pathways offer a foundation for further study into the mechanism behind HSPN progression.

## 2. Materials and Methods

### 2.1. Study Participants

All children were enrolled from the Pediatric Inpatient Department of the Affiliated Hospital, Harbin Medical University, from February 2013 to January 2017. HSP was diagnosed according to the criteria defined by the European League against Rheumatism/Paediatric Rheumatology International Trials Organization/Paediatric Rheumatology European Society (EULAR/PRINTO/PRES) [6]. HSPN was diagnosed with the presence of renal pathology during the first 6 months of HSP, manifesting as either hematuria and/or proteinuria [11]. The pathology grades of HSPN patients were obtained by renal biopsy. Under light microscopy, obvious differences were observed in the renal pathological sections from HSPN type 1 to type 3 (Supplementary Figure S1). Children with regular treatment for a chronic disease and those with urinary tract infections were excluded from this study. Collectively, this study included nine HSPN (3 × type 1, 3 × type 2, and 3 × type 3) as well as nine HSP patients that were age- and sex-matched (Table 1). The characteristics of study participants were collected from medical records. Additionally, another 9 patients (3 × type 1, 3 × type 2, and 3 × type 3) were included for validation purposes. All subjects provided written informed consent to participate in this study. This project was approved by Harbin Medical University's Ethical Review Committee. All methods were performed in accordance with the relevant guidelines and regulations.

### 2.2. Sample Collection

Plasma samples were collected from all participants before they received treatment. Whole blood samples (5 ml) were collected after 12 hours of fasting into an EDTA tube. It was then immediately centrifuged at 4000 × *g* for 10 min, and the supernatant was stored at -80°C until further analysis.

### 2.3. TMT-Based Proteomic Analysis

Each sample of serum (200 *μ*l) had highly abundant proteins removed by Bio-Rad ProteoMiner protein enrichment kits, and the total protein content for each sample was then quantified using a bicinchoninic acid (BCA) protein assay. This was followed by the reduction and alkylation, as well as the acetone precipitation of the sample, which was then resolved, tryptic digested, and labelled by tandem mass tag (TMT). Finally, the sample had SDC (sodium deoxycholate) removed and the peptides were desalinated. Reverse-phase high-performance liquid chromatography (RP-HPLC) was then performed. Peptides in each sample were separated by a nano-HPLC system, EASY-nLC1200, and were then detected using an online Q Exactive mass spectrometer (Thermo Finnigan). Separation of the sample was executed with a 90 min gradient at 300 nl/min flow rate. Gradient B is 5% for 3 min, 8-35% for 70 min, 35-45% for 15 min, 45-100% for 1 min, 100% for 2 min, 100-2% for 1 min, and 2% for 1 min. The original data obtained by liquid chromatography-tandem mass spectrometry (LC-MS/MS) were searched and quantified by MaxQuant (version 1.5.6.0). The protein database utilized was UNIPROT_HUMAN_2016_09, and the quantitative method employed was secondary reporter quantification with 10-labeled TMT, with labeled N-terminal polypeptide and Lys (K) sites. The product ion filter (PIF) was set to 0.75. The false discovery rate (FDR) was controlled at 0.01, and the proteins used in the quantitative analysis only included unmodified unique peptides. Simultaneously, an intensity-based absolute quantification (iBAQ) label-free quantitative approach was also carried out on the samples.

### 2.4. RNA-seq Analysis

Add 750 *μ*l TRIzol LS Reagent to 250 *μ*l plasma samples, homogenize, and incubate for 5 minutes. Add 0.2 ml of chloroform, and shake tubes vigorously and incubate them at 15-30°C for 2-3 minutes. Centrifuge the samples at 12,000 × *g* for 15 minutes at 4°C; the mixture was separated into three phases. RNA remained in the upper aqueous phase. Transfer the aqueous phase to a fresh tube and then add 0.5 ml of isopropyl alcohol, incubate samples at 15-30°C for 10 minutes, and centrifuge at 12,000 × *g* for 10 minutes at 4°C. Remove the supernatant and wash the RNA pellet once with 1 ml of 75% ethanol. Remove the supernatant and air-dry the RNA pellet for 5-10 minutes. Dissolve RNA in 85 *μ*l or less RNase-free water. Total RNA from each sample was quantified using the NanoDrop ND-1000 (Thermo Fisher Scientific, USA). 1-2 *μ*g total RNA was used to prepare the sequencing library in the following steps: firstly, total RNA was enriched by removing rRNA; secondly, RNA-seq library preparation used the KAPA Stranded RNA-Seq Library Prep Kit (Illumina, San Diego, USA), which incorporated dUTP into the second cDNA strand and rendered the RNA-seq library strand-specific. The completed libraries were qualified with Agilent 2100 Bioanalyzer (Agilent Technologies, USA) and quantified by the absolute quantification qPCR method. To sequence the libraries on Illumina HiSeq 4000, the barcoded libraries were mixed, denatured to single-stranded DNA in NaOH, captured on the Illumina flow cell, amplified in situ, and subsequently sequenced for 150 cycles for both ends on the Illumina HiSeq instrument.

### 2.5. Validation of the Transcriptomic and Quantitative Proteomic Data

In order to validate the high-throughput results of the transcriptomic and proteomic analyses, a total of 10 mRNAs and 10 proteins found to be differential expressed between different comparison groups were chosen for validation. Their expression levels were measured by either quantitative reverse transcription polymerase chain reaction (qRT-PCR) (mRNAs) or Parallel Reaction Monitoring (PRM) (proteins) [16]. The detailed experimental procedure of PRM was introduced in Supplementary Materials. The validation cohort included an additional nine HSPN (3 × type 1, 3 × type 2, and 3 × type 3) patients from which new serum samples were taken and assayed.

### 2.6. Bioinformatics and Statistical Analysis

After data normalization, Student's *t*-tests were performed to obtain the significance of the differential gene expression. The mRNAs and proteins were regarded as differentially expressed when found with a *P* value < 0.05 and a fold change greater than 1.5-fold (ratio A/B > 1.5 or ratio A/B < 2/3). Principal component analysis (PCA) was performed to visualize the separation among the different tested groups. A correlation analysis of the differentially expressed mRNAs and proteins (DEGs) was conducted using the mixOmics package in R [17]. The clusterProfiler package was used to conduct the functional annotation of the DEGs for both Gene Ontology (GO) terms and Kyoto Encyclopedia of Genes and Genomes (KEGG) pathways [18]. Benjamini-Hochberg-adjusted *P* < 0.05 (FDR) was used as the threshold to determine the significance of each of the pathways identified. All statistical analyses were performed in the R platform (version 3.4.3). Heat maps were generated in GraphPad Prism (version 7.0). A two-sided *P* < 0.05 was established as the level of statistical significance for all tests.

## 3. Results

### 3.1. Differential Transcriptomic and Proteomic Profile

#### 3.1.1. Differentially Expressed mRNAs and Proteins

**Figure 1:**
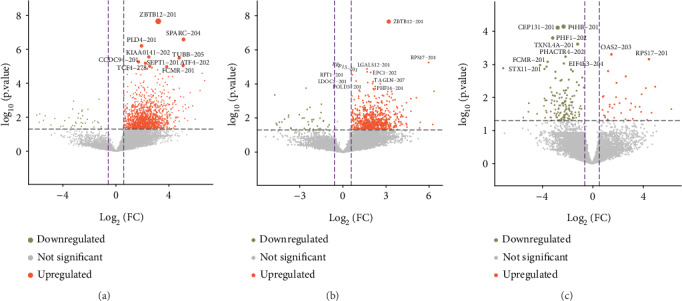
The volcano plots of the differentially expressed mRNAs identified between different groups. (a) Type 1 vs. type 2. (b) Type 1 vs. type 3. (c) Type 2 vs. type 3. Downregulated (khaki) and upregulated (orange red) mRNAs are indicated. mRNAs found to be not significantly altered between the groups are displayed in gray. FC: fold change. The top 10 mRNAs according to their *P* value ranking are listed. The circle size is proportional to the *P* value.

**Figure 2:**
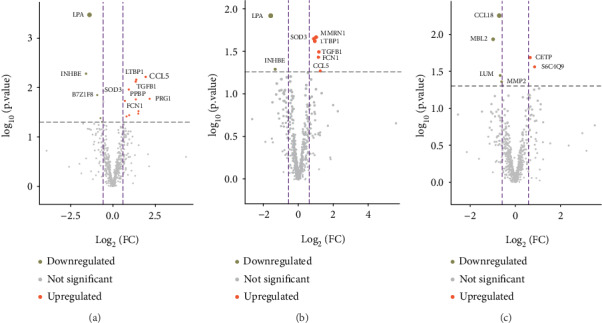
The volcano plots of the differentially expressed proteins between different groups. (a) Type 1 vs. type 2. (b) Type 1 vs. type 3. (c) Type 2 vs. type 3. Downregulated (khaki) and upregulated (orange red) proteins are indicated. Proteins found to be not significantly altered between the groups are displayed in gray. FC: fold change. The top 10 proteins according to their *P* value ranking are listed. The circle size is proportional to the *P* value.

A total of 24,493 mRNAs (13,327 genes) and 592 proteins (4793 peptides) were quantitated (Supplementary Table S1). Based on the criteria, a total of 2315 mRNAs were found to be differentially expressed between different types of HSPN, with 2094 upregulated and 221 downregulated (Supplementary Table S2). Furthermore, 30 proteins were differentially expressed between the different types of HSPN, with 19 upregulated and 11 downregulated (Supplementary Table S3). The DEGs, mRNAs and proteins, between the contrasting groups can be seen in Figures 1 and 2, respectively. As seen in Supplementary Figure S2, an obvious separation between type 1 and type 2, as well as type 1 and type 3, is noted.

#### 3.1.2. The Patterns of DEGs Change from Type 1 to Type 3

Further 58 mRNAs and one protein were identified to be concurrently differentially expressed between type 1 and type 2, as well as type 2 and type 3. Among them, most of the DEGs (51) were found to first be significantly downregulated in early HSPN (type 1 to type 2) and then significantly upregulated in the later stages of HSPN (type 2 to type 3). However, only seven DEGs were found to first be significantly upregulated in early HSPN and significantly downregulated in the later stages. Furthermore, it is worth noting that RPS17-201 was found to be continuously downregulated throughout HSPN progression (type 1 to type 3) (Supplementary Table S4).

### 3.2. Correlation Analysis of Transcriptomic and Proteomic Data

A correlation analysis of DEGs between different types of HSPN was performed. The sample scatterplot seen in Supplementary Figure S3A shows that the first latent components of each of the “omics” data sets were highly correlated between each other (*r* = 0.96) and that these components were able to discriminate between type 1 and type 2, as well as type 1 and type 3 patients. A signature was noted for the first two components of the two “omics” data sets, with 20 and 10 mRNAs and 10 and three proteins, respectively. Correlation circle plots, as seen in Supplementary Figure S3B, further highlighted correlations between each selected feature and its associated latent component. A circosPlot displays the different types of selected features (30 mRNAs and 13 proteins) on a circle. The links between or within two “omics” data sets indicate a strong positive or negative correlation, and as can be seen in Supplementary Figure S3C, there was a strong correlation between the mRNA and protein observed.

### 3.3. Pathway Enrichment Analysis

A total of 309 significant pathways were enriched with the upregulated DEGs found between the different types of HSPN, including 219 biological process terms, eight molecular function terms, 76 cellular component terms, and six KEGG pathways (Supplementary Table S5). They were mainly classified into inflammation and immunity, cell apoptosis, platelet activation and blood coagulation, epidermal growth factor (EGF), pathways related to the repair of renal injury, and cytokines associated with tumors, apoptosis, inflammation, and kinases. A total of 76 significant pathways were enriched with the downregulated DEGs identified between the different types of HSPN, including 28 biological process terms, five molecular function terms, 39 cellular component terms, and four KEGG pathways (Supplementary Table S5). They were mainly classified into inflammation and immunity and platelet activation and blood coagulation.

**Figure 3:**
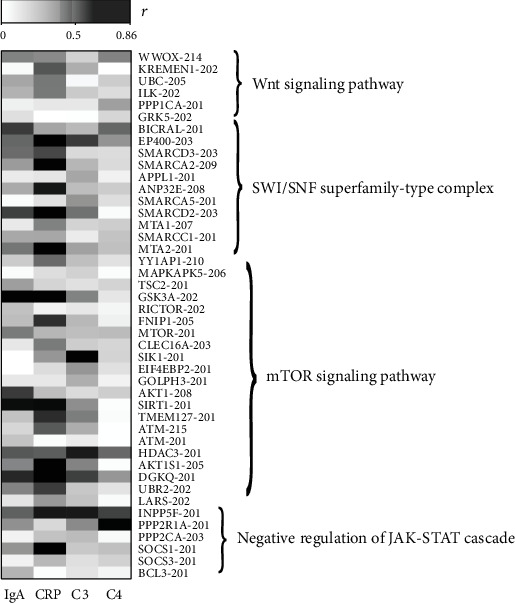
Correlation analysis between differentially expressed genes in the four pathways and clinical biochemical indicators.

We further identified pathways which were only related to HSPN progression; the identification steps were as follows: Firstly, pathways which were enriched with DEGs between the different types of HSPN were identified. Secondly, those pathways which were enriched with DEGs between HSP and HSPN were excluded (Supplementary Table S8 and Supplementary Table S9). Thirdly, those pathways in which most differentially expressed genes were closely related to clinical biochemical indicators from the remaining pathways were identified. Ultimately, four pathways (negative regulation of the JAK-STAT cascade, the mTOR signaling pathway, the SWI/SNF superfamily-type complex, and the Wnt signaling pathway) were identified. The basic information of differentially expressed genes in four pathways is shown in Supplementary Table S10. Most differentially expressed genes in four pathways were closely related to clinical biochemical indicators (Figure 3); their detailed correlation coefficients are shown in Supplementary Table S6. The results indicated that these four pathways may play an important role in the progression of HSPN.

### 3.4. Validation of the Transcriptomic and Proteomic Data

**Figure 4:**
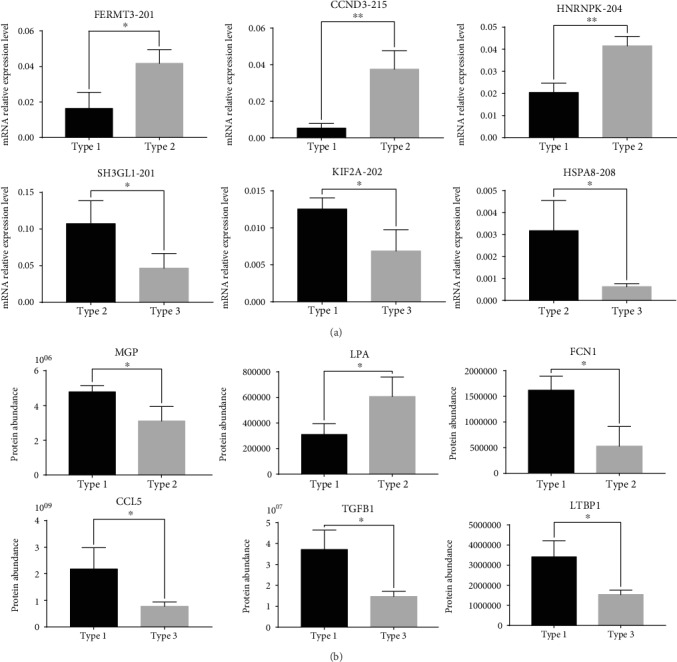
Validation of transcriptomic and proteomic data. ^∗^*P* < 0.05, ^∗∗^*P* < 0.01: (a) mRNA; (b) protein.

Based on the fold changes of the DEGs, a total of 10 mRNAs and 10 proteins which were found to be differentially expressed between the various comparison groups were chosen (Supplementary Table S7). Their expression levels were measured and validated by qRT-PCR (mRNAs) and PRM (proteins) methods, respectively. As shown in Figure 4(a), qRT-PCR results for six of the 10 selected mRNAs identified in the differential analysis were found to be consistent with the transcriptomic results. Of the 10 selected proteins identified in the differential analysis, six were successfully validated and found to be consistent with the quantitative proteomic data (Figure 4(b)). Taken together, these results demonstrate the reliability of the “omics” data generated in this study.

## 4. Discussion

In this study, a multiple genome-wide approach, including transcriptomics and proteomics, was integrated together to identify significant novel pathways for HSPN progression. It was found that a total of 2315 mRNAs and 30 proteins were differentially expressed between different types of HSPN (Supplementary Table S2 and Supplementary Table S3). In particular, these mRNAs and proteins were ranked according to their *P* values (in ascending order), and the functions of the top 10 mRNAs and proteins were searched in GeneCards and UniProt databases. They mainly were found to be involved in immunity, apoptosis, platelet and coagulation, and tumor necrosis, consistent with the pathway enrichment results, and those functions were related to nephritis and renal injury [10, 15, 19]. It is worth noting that most proteins were differentially expressed between different types of HSPN while their corresponding mRNAs were not differentially expressed between those groups (Supplementary Table S2 and Supplementary Table S3). Our results found that there existed the mechanisms of the posttranscriptional regulation of gene expression in HSPN progression. Gan et al. also revealed multiple posttranscriptional regulatory mechanisms of mouse spermatogenesis by integrating proteomic and transcriptomic analyses [20].

We further identified 58 mRNAs and one protein concurrently differentially expressed between type 1 and type 2 and type 2 and type 3 HSPN patients. The DEGs found to be active throughout HSPN progression may be potential biomarker candidates for HSPN classification. Of these, RPS17-201 is of most promise with it being found to be continuously downregulated throughout HSPN disease. RPS17 encodes a ribosomal protein, involved in the generation of serum IgA [15]. Furthermore, it was found that most DEGs were only differentially expressed in either early HSPN or late HSPN (Supplementary Table S2 and Supplementary Table S3, respectively). This implies that these genes and proteins are only active during a single stage of HSPN and are then relatively silent during another stage. This investigation of the patterns of DEG changes from type 1 to type 3 indicates that HSPN progression undergoes various stages wherein different genes and proteins play various roles in various and specific periods of the disease. This finding is consistent with previous studies [2, 3].

A total of 385 significant pathways were found to be enriched with DEGs between the different types of HSPN, and they were mainly classified into inflammation and immunity, cell apoptosis, platelet activation and blood coagulation, EGF, pathways related to the repair of renal injury, and cytokines associated with tumors, apoptosis, inflammation, and kinases. Four pathways—negative regulation of the JAK-STAT cascade, the mTOR signaling pathway, the SWI/SNF superfamily-type complex, and the Wnt signaling pathway—were closely related to clinical biochemical indicators, which indicated that they may play an important role in the progression of HSPN.

Many proinflammatory and proapoptotic cytokines transmit signals through the JAK-STAT signaling pathway, and these include interleukins, granulocyte/macrophage colony-stimulating factors, and TNF-*α* [21, 22]. HSPN is a small-vessel form of the autoimmune vasculitis caused by IgA1-mediated inflammation [4], and its progression is closely related to the aggravation of the renal inflammatory response [2]. Apoptosis is an important mechanism to regulate and prevent inflammatory injury [23]. This study is the first to report that the JAK-STAT signaling pathway is active during the progression of HSPN. Previously, the JAK/STAT pathway has been shown to play an important role in the development of obstructive nephropathy [24], diabetic nephropathy [25], and acute kidney injury [26]. Another pathway identified in this study, the Wnt signaling pathway, has been noted to regulate many biological processes, including proliferation, migration, invasion, and apoptosis [27]. Previously, He et al. have reported that the Wnt signaling pathway may be involved in the modulation of HSPN pathogenesis [10]. In this study, this pathway was found to be active during the progression of HSPN from type 2 to type 3 (Supplementary Table S5), a period when renal injury can be relatively serious. Kawakami et al. have previously indicated that the Wnt signaling pathway is involved in the repair of the renal tubular epithelial cells after renal injury [28]. Therefore, due to the Wnt signaling pathway most likely playing an important role in alleviating renal injury, it was found to be active in the development of HSPN. Additionally, this study showed that the mTOR signaling pathway is active during the progression of HSPN from type 1 to type 2 (Supplementary Table S5). The mTOR signaling pathway participates in the regulation of many cellular functions, including proliferation, growth, differentiation, and apoptosis [29]. Previously, Zhang et al. have reported that the mTOR signaling pathway is involved in the proliferation of mesangial cells due to IgA1 isolated from HSP patients, most likely related to the mesangial injury of HSPN [30]. Furthermore, Xu et al. have shown that the mTOR signaling pathway is activated in renal tissues of children with immunoglobulin A nephropathy [31]. Another classification identified in this study was the SWI/SNF superfamily-type complex, and it was found to be active during the progression of HSPN from type 1 to type 2 (Supplementary Table S5). It has been indicated that the p53 protein-dependent apoptosis is suppressed by the chromatin remodeling factor SMARCD1 [32]. Additionally, Hu et al. found that SWI/SNF-associated chromatin remodeling was related to the inflammatory response in macrophages [33]. As described above, HSPN progression is closely related to the aggravation of renal inflammation and also associated with apoptosis [4, 23]. This study is the first to report that the SWI/SNF superfamily-type complex is active in the progression of HSPN. In addition, this study revealed that platelet activation and blood coagulation as well as EGF were both active in the progression of HSPN. Of note, inflammation is associated with platelet coagulation function, and proinflammatory cytokines are capable of activating the coagulation system [34]. Furthermore, EGF can promote the proliferation and repair of renal tubular epithelial cells when the kidney is injured [35].

The results of this study reveal that HSPN progression may be related to the inhibition of inflammation, promotion of apoptosis, and repair of renal injury. It is known that the renal inflammatory response aggravates and activates the coagulation system during the progression of HSPN [2]. Furthermore, proapoptotic cytokines, such as TNF-*α*, can induce apoptosis of inflammatory cells through the JAK-STAT signaling pathway and limit the expansion of inflammation. Apoptosis also occurred in the renal tubular cells and leads to tubular cell loss and tubular dysfunction [36]. Additionally, aggravated renal injury triggers the process of repairing and remodeling of the damaged tubules and promotes their return to normal structural and functional states [36] through EGF and the Wnt and mTOR signaling pathways. Therefore, we hypothesize that this is a possible underlying molecular mechanism of HSPN progression, and as such this study provides important clues for finding novel therapeutic targets in the future. Undoubtedly, further research is required to confirm this hypothesis.

To note, there are limitations to this study. The major limitation is the small sample size, but this is due to the requirement of a renal biopsy for the diagnosis of HSPN classification in this study. The number of pediatric patients who accepted to have a renal biopsy was rare, which resulted in fewer samples. Furthermore, we did not include kidney tissues of the patients tested or use an animal model to verify our results *in vivo*.

In conclusion, an integrated transcriptomic and proteomic analysis was performed to identify significant novel pathways for HSPN progression. A total of 2315 mRNAs and 30 proteins were differentially expressed between different types of HSPN. Additionally, 58 mRNAs and one protein were found to continuously change during HSPN development and therefore could be used as potential markers for various stages of HSPN progression. A total of 385 significant pathways were enriched with DEGs found between different types of HSPN. The negative regulation of the JAK-STAT cascade, the mTOR signaling pathway, the SWI/SNF superfamily-type complex, and the Wnt signaling pathway were found to be closely related to HSPN progression. Therefore, it seems that HSPN progression is mainly due to the inhibition of inflammation, promotion of apoptosis, and repair of renal injury. This is the first study to integrate both transcriptomics and proteomics into a single study to identify significant novel pathways for HSPN progression using samples from different stages of HSPN. The “omics” data generated by this study may aid in continuing the understanding of the molecular mechanisms of HSPN progression and constitute a solid base for further research in the future.

## Figures and Tables

**Table 1:** The demographic and clinical characteristics of patients in this study.

	HSP									HSPN (type 1)			HSPN (type 2)			HSPN (type 3)		
Pathological type	—	—	—	—	—	—	—	—	—	Ia	Ib	Ib	IIb	IIa	IIb	IIIa	IIIa	IIIb
Patient ID	1	2	3	4	5	6	7	8	9	1	2	3	1	2	3	1	2	3
Sex	1^a^	2	2	1	1	2	1	1	2	1	1	2	2	1	1	1	1	2
Man/woman	5/4									6/3								
Age	7	15	13	6	13	11	10	7	6	11	10	5	9	10	16	8	9	10
Mean age	9.78									9.78								
Arthralgias and/or arthritis^b^	1	0	2	0	1	1	0	1	0	1	1	0	2	0	0	1	1	1
Bowel angina and/or gastrointestinal bleeding	2	0	0	0	1	2	0	0	1	1	0	1	1	0	1	1	2	1
Proteinuria/hematuria	0	1	0	0	0	0	1	0	0	2	2	2	3	2	3	3	3	3
IgA (g/l)	2.53	1.67	2.54	1.65	2.18	1.21	1.98	1.94	1.96	1.02	1.19	2.45	1.72	3.35	4.48	2.65	3.00	7.16
Mean IgA	1.96									1.55			3.18			4.27		
CRP (mg/l)	1.51	2.58	1.89	0.73	0.79	3.14	1.77	1.98	1.56	1.67	1.87	1.43	6.32	8.38	8.42	11.45	15.05	20.80
Mean CRP	1.77									1.66			7.71			15.77		
C3 (g/l)	1.01	0.86	0.79	1.03	0.96	0.86	0.92	1.12	0.72	0.95	1.12	1.08	1.14	1.16	1.12	1.04	1.46	1.16
Mean C3	0.92									1.05			1.14			1.22		
C4 (g/l)	0.15	0.22	0.19	0.12	0.22	0.35	0.23	0.21	0.20	0.12	0.27	0.26	0.33	0.19	0.23	0.22	0.26	0.19
Mean C4	0.21									0.22			0.25			0.22		

^a^1 and 2 denote boy and girl, respectively. ^b^Joint: 0 = no symptoms; 1 = pain and/or slight swelling; 2 = pain and/or moderate swelling; 3 = pain and/or severe swelling; GI: 0 = no symptom; 1 = slight pain and/or occult stool blood (OSB) (+); 2 = moderate pain and/or OSB (+2, +3); 3 = severe and/or melena; kidney: 0 = no proteinuria; 1 = proteinuria (+) and/or hematuria (+); 2 = proteinuria (2+, 3+) and/or hematuria (2+, 3+); 3 = proteinuria (>3+) and/or hematuria (>3+).

## Data Availability

The raw data cannot be shared at this time as the data also forms part of an ongoing study.

## Supplementary Materials

Supplementary Materials .Supplementary Figure S1: images of renal pathological sections of HSPN patients. (A) Type 1. (B) Type 2. (C) Type 3. Supplementary Figure S2: PCA score plots for discriminating samples within the different groups. Supplementary Figure S3: correlation analysis of differentially expressed mRNAs and proteins between different types of HSPN. Supplementary Table S1: list of quantitated 24,493 mRNAs and 592 proteins. Supplementary Table S2: list of differentially expressed mRNAs between different HSPN types. Supplementary Table S3: list of differentially expressed proteins between different HSPN types. Supplementary Table S4: list of DEGs which were differentially expressed between HSPN type 1 and HSPN type 2 and HSPN type 2 and HSPN type 3. Supplementary Table S5: the significant pathways enriched by DEGs between different HSPN types. Supplementary Table S6: the correlation coefficients between differentially expressed genes in four pathways and clinical biochemical indicators. Supplementary Table S7: list of mRNAs and proteins selected for validation. Supplementary Table S8: the significant pathways enriched by downregulated DEGs between HSP and HSPN. Supplementary Table S9: list of differentially expressed mRNAs between HSP and HSPN. Supplementary Table S10: the differentially expressed genes in four pathways negative regulation of the JAK-STAT cascade and so on.
