# Supplementary figures and images for: An Integrated Transcriptomic and Proteomic Analysis Identifies Significant Novel Pathways for Henoch-Schönlein Purpura Nephritis Progression
Source: Biomed Res Int. 2020 Jun 19;2020:2489175. doi: 10.1155/2020/2489175 (PMC7322592; doi:10.1155/2020/2489175)

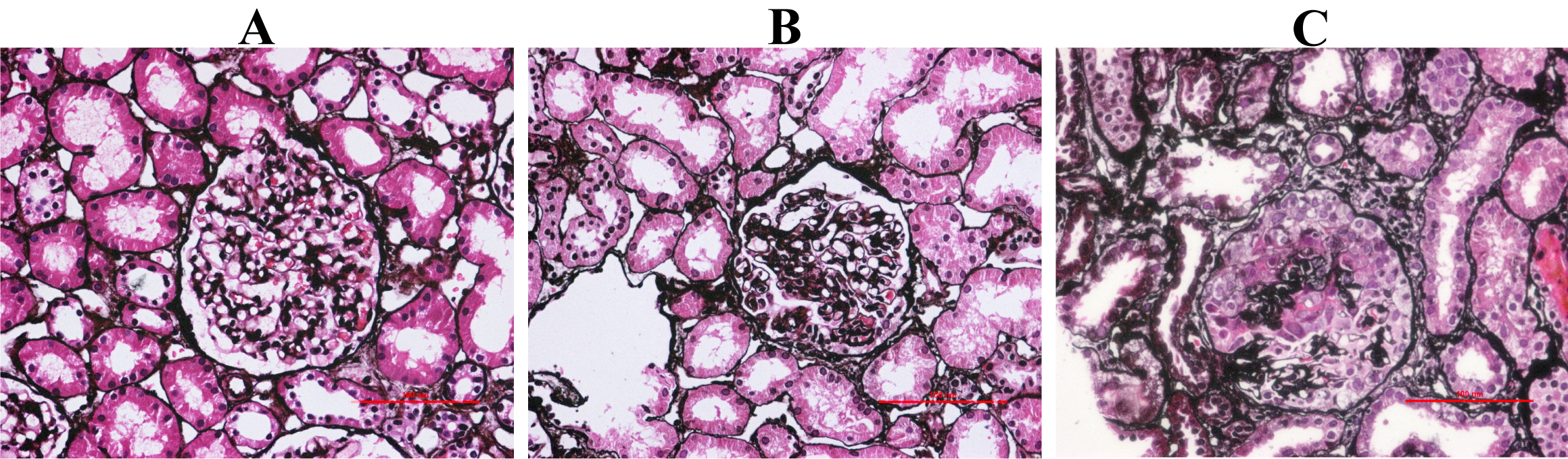

Supplement: Supplementary Materials — .Supplementary Figure S1: images of renal pathological sections of HSPN patients. (A) Type 1. (B) Type 2. (C) Type 3. Supplementary Figure S2: PCA score plots for discriminating samples within the different groups. Supplementary Figure S3: correlation analysis of differentially expressed mRNAs and proteins between different types of HSPN. Supplementary Table S1: list of quantitated 24,493 mRNAs and 592 proteins. Supplementary Table S2: list of differentially expressed mRNAs between different HSPN types. Supplementary Table S3: list of differentially expressed proteins between different HSPN types. Supplementary Table S4: list of DEGs which were differentially expressed between HSPN type 1 and HSPN type 2 and HSPN type 2 and HSPN type 3. Supplementary Table S5: the significant pathways enriched by DEGs between different HSPN types. Supplementary Table S6: the correlation coefficients between differentially expressed genes in four pathways and clinical biochemical indicators. Supplementary Table S7: list of mRNAs and proteins selected for validation. Supplementary Table S8: the significant pathways enriched by downregulated DEGs between HSP and HSPN. Supplementary Table S9: list of differentially expressed mRNAs between HSP and HSPN. Supplementary Table S10: the differentially expressed genes in four pathways negative regulation of the JAK-STAT cascade and so on. [file 2489175.f1.zip › Supplementary Figure S1.tif]

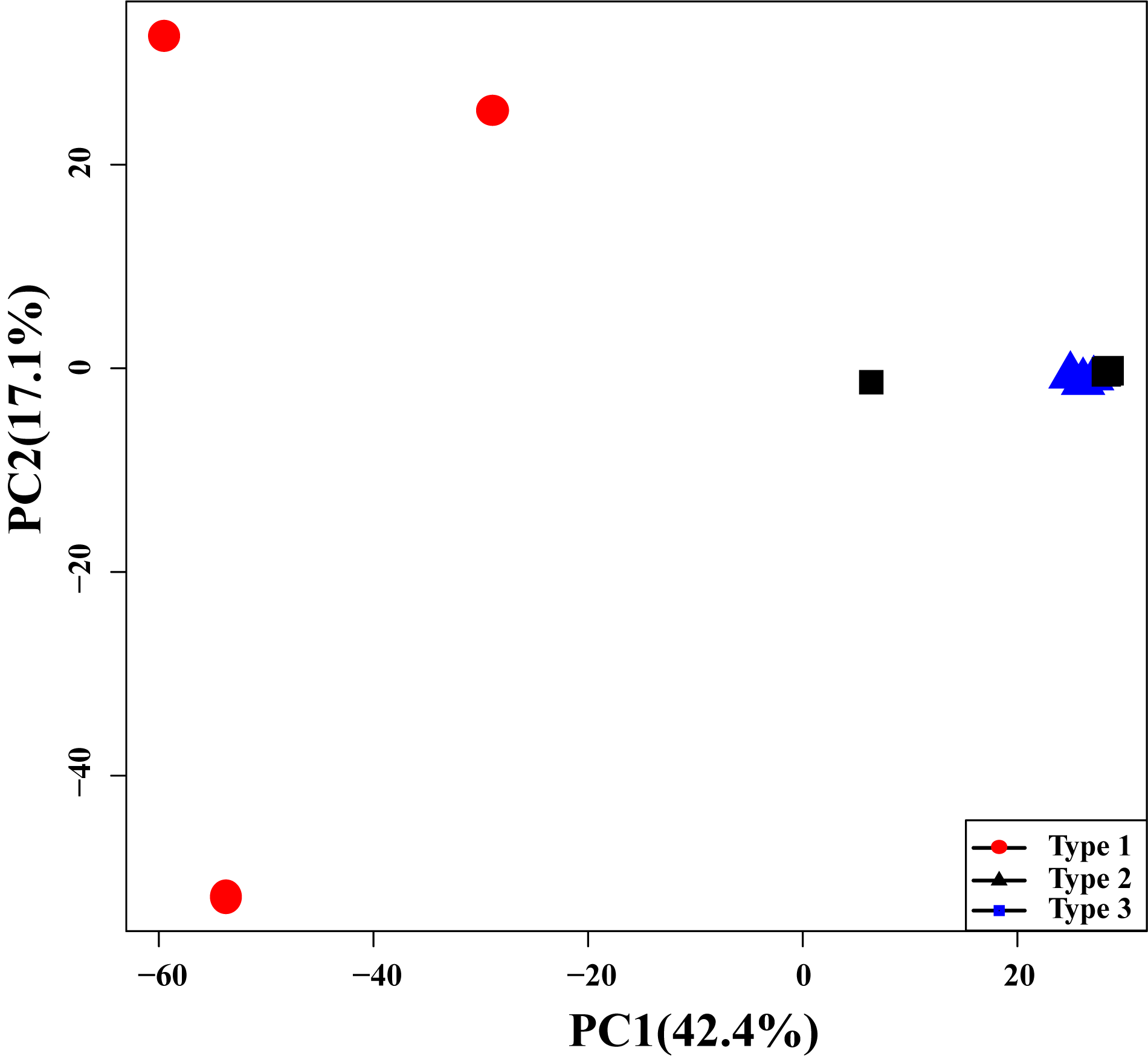

Supplement: Supplementary Materials — .Supplementary Figure S1: images of renal pathological sections of HSPN patients. (A) Type 1. (B) Type 2. (C) Type 3. Supplementary Figure S2: PCA score plots for discriminating samples within the different groups. Supplementary Figure S3: correlation analysis of differentially expressed mRNAs and proteins between different types of HSPN. Supplementary Table S1: list of quantitated 24,493 mRNAs and 592 proteins. Supplementary Table S2: list of differentially expressed mRNAs between different HSPN types. Supplementary Table S3: list of differentially expressed proteins between different HSPN types. Supplementary Table S4: list of DEGs which were differentially expressed between HSPN type 1 and HSPN type 2 and HSPN type 2 and HSPN type 3. Supplementary Table S5: the significant pathways enriched by DEGs between different HSPN types. Supplementary Table S6: the correlation coefficients between differentially expressed genes in four pathways and clinical biochemical indicators. Supplementary Table S7: list of mRNAs and proteins selected for validation. Supplementary Table S8: the significant pathways enriched by downregulated DEGs between HSP and HSPN. Supplementary Table S9: list of differentially expressed mRNAs between HSP and HSPN. Supplementary Table S10: the differentially expressed genes in four pathways negative regulation of the JAK-STAT cascade and so on. [file 2489175.f1.zip › Supplementary Figure S2.tif]
